# Occupational Participation Among Older Adults During the COVID-19 Pandemic

**DOI:** 10.1177/00084174241287297

**Published:** 2024-10-03

**Authors:** Samantha A. Oostlander, Camille Joanisse, Michael S. Mulvey, Sarah Fraser, Martine Lagacé, Louise Bélanger-Hardy, Linda Garcia, Annie Robitaille, Margaret Gillis, Jill Courtemanche, Tracey L. O’Sullivan

**Keywords:** Canadian model of occupational participation, Disaster risk reduction, Occupational possibilities, Occupational disruption, Modèle canadien de la participation occupationnelle, possibilités occupationnelles, réduction des risques de catastrophe, rupture occupationnelle

## Abstract

**Background**. The COVID-19 pandemic led to abrupt occupational disruption for all people. However, some populations, like older adults, were disproportionately impacted particularly in the earlier waves. **Purpose.** The purpose of this study was to explore and understand how the occupational participation of community-dwelling older adults was experienced during the COVID-19 pandemic, using the Canadian Model of Occupational Participation (CanMOP) to contextualize findings. **Method.** Sixty-seven older adults participated in semi-structured interviews from September 2020 to May 2021, 37 of which also participated in a follow-up interview one-year later. **Findings.** Using reflexive thematic analysis, four themes were generated: (1) experiences of loss are complex and layered for older adults, (2) technology as a medium for occupational participation, (3) risk perception influences return to occupation, and (4) age-related challenges for older adults resuming volunteer work. **Conclusion.** Increasing frequency and severity of influenza pandemics and other disasters are a global concern, and OTs can use their skillsets to foster participation and expand occupational possibilities for older adults. The CanMOP was a helpful tool to understand the nuances underlying the participation of older adults in this context.

## Résumé

**Description.** La pandémie de COVID-19 a entraîné une brusque rupture occupationnelle pour tout le monde. Cependant, certaines populations, comme les personnes âgées, ont été touchées de façon disproportionnée, particulièrement lors des premières vagues. **But**. Le but de cette étude était d’explorer et de comprendre comment la participation occupationnelle des personnes âgées résidant dans la communauté a été vécue pendant la pandémie de COVID-19, en utilisant le Modèle canadien de la participation occupationnelle (MCPO) pour contextualiser les résultats. **Méthodologie.** Soixante-sept personnes âgées ont participé à des entrevues semi-structurées de septembre 2020 à mai 2021, dont 37 qui ont également participé à une entrevue de suivi un an plus tard. **Résultats.** Une analyse thématique réflexive a été réalisée, et quatre thèmes ont été générés : 1) Les expériences de perte sont complexes et stratifiées chez les personnes âgées; 2) La technologie comme moyen de favoriser la participation occupationnelle; 3) La perception du risque influence le retour à l’occupation; et 4) Les défis liés à l’âge pour les personnes âgées qui reprennent le bénévolat. **Conclusion.** L’augmentation de la fréquence et de la gravité des pandémies d’influenza et d’autres catastrophes est un enjeu mondial, et les ergothérapeutes peuvent mobiliser leurs compétences pour favoriser la participation et élargir les possibilités occupationnelles des personnes âgées. Le MCPO a été un outil utile pour comprendre les nuances sous-jacentes à la participation des personnes âgées dans ce contexte.

## Introduction

The focus of occupational therapists (OTs) is to support participation in meaningful occupations—from everyday to extraordinary activities that people want, need or have to do to live their lives (Laliberte Rudman et al., 2022). More frequent and severe disasters, such as influenza pandemics, can disrupt occupational participation for all people, as seen during the COVID-19 pandemic (United Nations Office for Disaster Risk Reduction [UNDRR], 2021). However, some groups of people experience more disproportionate impacts than others; for example, during the earlier waves of the COVID-19 pandemic, older adults were at greater risk for fatality and other health complications than other age demographics (Centers for Disease Control and Prevention [CDC], 2023). Reduced social supports (formal and informal), isolation, and limited income further challenged the quality of life of older adults during this time (Mental Health Commission of Canada, 2021). Understanding the influence of the COVID-19 pandemic on the occupational participation of older adults can help to inform how OTs can support the well-being of this population for future pandemics.

Scholarly literature indicates that older adulthood is often marked by occupational disruption—changes to patterns of participation in daily occupations (Whiteford, 2000). Examples of factors that may lead to disruption include, retirement from the workforce, navigating chronic health conditions, social losses, and responsibilities like caregiving (Nizzero et al., 2017; Walker & McNamara, 2013). Studies have reported that during the COVID-19 pandemic, older adults experienced challenges accessing online activities, grappled with emotional distress, and experienced changes in mood and sense of belonging within their communities (Carlsson et al., 2022; Fristedt et al., 2022; Rotenberg et al., 2021; Vesnaver et al., 2023). Public health restrictions implemented during the pandemic, like physical distancing, are being described as a paradox in relation to older adults; intended protection from illness also led to negative implications for health and well-being stemming from social isolation, for example (Tyrrell & Williams, 2020).

While all people had to navigate changes to daily routines due to public health restrictions, older adults also faced additional challenges like ageism—the stereotyping, prejudice, and discrimination of individuals based on age—which embeds itself implicitly into institutions and often goes unchallenged (Braithwaite, 2002; Butler, 1969; Lagacé et al., 2024). Studies have reported that ageism towards older adults intensified and there was a rise in intergenerational tension during the pandemic; for example, the view that restrictive public health measures, such as lockdowns, benefited older adults at the expense of younger people (Ayalon et al., 2021; Fraser et al., 2020; Levy et al., 2022).

Ageism can lead to experiences of stigma, exclusion and isolation, and restricts occupational possibilities (Laliberte Rudman, 2015)—“*ways and types of doing that come to be viewed as ideal and possible with a specific socio-historical context, and that come to be promoted and made available within that context*” (Laliberte Rudman, 2010, p. 55). The rise in intergenerational tensions stemming from ageism was found to contribute to hostility and hindered the social participation of older adults in society (Ayalon et al., 2021; Levy et al., 2022; Swift & Chasteen, 2021). Through their training, OTs are well situated to identify and understand how social factors, like ageism, may influence occupational participation (Egan & Restall, 2022).

The *Canadian Model of Occupational Participation* (CanMOP) is a guiding model which holds the concept of occupational participation as its central focus (Egan & Restall, 2022). Occupational participation is “*having access to, initiating, and sustaining valued occupations within meaningful relationships and contexts*” (Egan & Restall, 2022, p. 76). Exploration of purpose and meaning rests on the assumption that people participate in occupations to meet needs for safety, survival, autonomy, competency, and relationships (Egan & Restall, 2022). The occupational possibilities giving rise to participation are further shaped by the environment's dynamic macro, meso, and micro factors.

The purpose of this study was to explore and understand how the occupational participation of community-dwelling older adults was experienced during the COVID-19 pandemic. The objective was to describe older adults’ experiences of participation during the pandemic using the CanMOP to situate our understanding of occupational participation. Our findings add to the growing literature on older adults’ experiences of the COVID-19 pandemic using OT theory, and provide useful insights for OTs who work with older adults.

## Methods

This study is embedded within a larger research program exploring how older adults experienced the pandemic and subsequent public health restrictions in Canada. For the research program, data were collected at two time-points via semi-structured interviews. This paper uses data from the broader research program for a secondary analysis in which a narrative approach was used to understand how the pandemic influenced the occupational participation of community-dwelling older adults living in Canada (Riessman, 2008). The University of Ottawa research ethics committee (H-04-22-7965) approved use of the data for secondary analysis on April 21, 2022. In the section below, we describe the overall process for data collection and identify how data was analyzed for this paper.

### Data Collection

While older adulthood does not begin at a universally agreed upon age, the World Health Organization (WHO) indicates older adults are people aged 60 years and older (WHO, 2020). We used this as a guide for the inclusion criteria which were as follows: aged 60 years and older, living in a community setting in Canada, and able to communicate in French or English. Recruitment was achieved through purposive and snowball sampling; the research team circulated recruitment posters via email through team members’ personal and professional networks, and asked participants to do the same with people in their social circles. The research team attempted to achieve maximum variation of sociodemographic characteristics to capture diverse perspectives; potential participants contacted and were screened by the principal investigator (T.O.).

Data from two time-points during the pandemic was collected to capture diverse experiences. Initial interviews were completed with *n* = 67 older adults from September 2020 to May 2021. Approximately one year later, from January 2022 to August 2022, *n* = 37 older adults from the same sample also completed a follow-up interview. The time lapse between the two interviews was intended to allow the research team to capture evolving experiences. Interviews were conducted via telephone by multiple team members (T.O., S.O., C.J., M.L., L. B-H.) and lasted 30–60 min. Participants provided informed consent to audio-record the interviews.

For both interviews, the same interviewers asked open-ended questions covering various topics relevant to the broader research program, for example, social connection, daily activities, and health and well-being. Interviews were transcribed verbatim by the research team. Reflections about the interview process and high-level analysis of the data led to iterative changes to the interview guides which improved question clarity during the data collection process. Table 1 shows some examples of questions used in the interviews to solicit narratives from participants relevant to this particular secondary analysis of data from the broader research program.

**Table 1:** Examples of Semi-Structured Interview Questions, Prompts, and Probes Posed to Participants for Both Interviews

**Questions posed to participants**
Can you describe your daily life and the activities that you took part in before the pandemic? Or since last we spoke? Are there any activities that you are missing during the pandemic with restrictions in place? If so, can you tell us something about it, what do you miss? Who did, or do, you do these activities with? What do these activities mean to you? If you can’t do this activity, what would you need to be able to start doing it again? Are there any new activities that you’ve started doing during the pandemic? Or since last we spoke?
**Prompts and probes used to clarify discussions as needed**
Tell me more about that? Can you please explain that further? Can you give an example?

### Data Analysis

**Figure 1.**
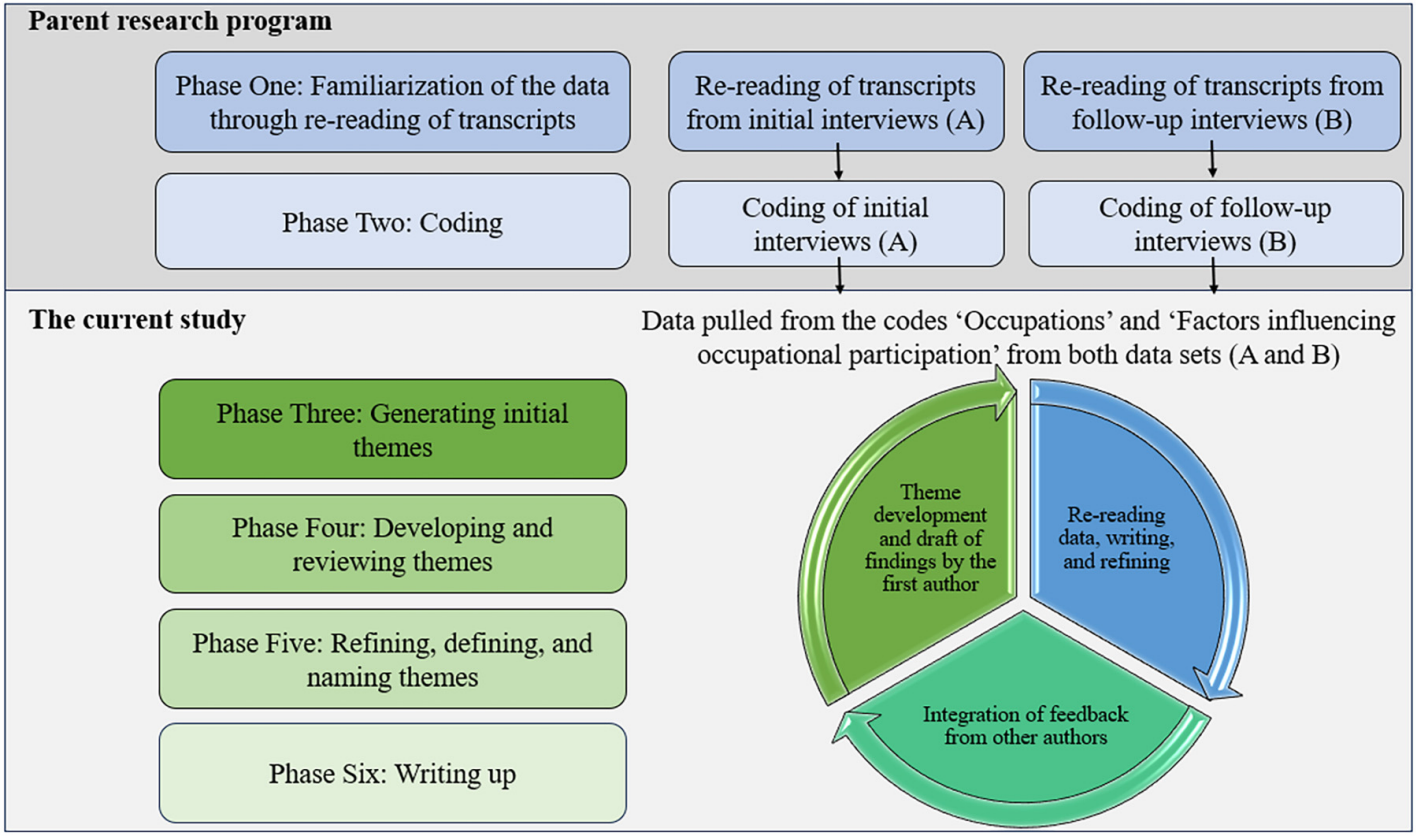
Schematic of the data analysis process using the iterative phases of reflexive thematic analysis described by Braun and Clarke (2022).

All interviews conducted were analyzed regardless of whether participants completed an initial interview only or both an initial and follow-up interview. Reflexive Thematic Analysis was completed to develop an understanding of older adults’ experiences as expressed through stories told (Braun & Clarke, 2022). Our process moving through the phases of reflexive TA are displayed in a schematic in Figure 1. Phases 1 and 2, familiarization and coding, were completed separately for each data set (initial and follow-up interview). In phase 1, three authors (S.O., C.J., T.O.) familiarized themselves with the data by re-reading the transcripts. In phase 2, the same authors inductively developed a coding grid and tested it on three transcripts before continuing to code independently. NVivo^12^ software facilitated coding (QSR International Pty Ltd., 2020).

From the main codebook, two codes “Occupations” and “Factors influencing occupational participation” from both interviews captured content used for theme generation in this study. The third, fourth, and fifth phases, which involve generating, reviewing, and defining and naming themes were completed by the first author (S.O.) in an iterative process using data from both data sets. The development of themes was influenced by CanMOP (Egan & Restall, 2022). The first author used the two essential components of the model (1—the purpose and meaning associated with participation, and 2—occupational possibilities afforded by the participants unique context) to support theme generation. Themes were mapped onto CanMOP, and are displayed in a thematic map in the results section. Team members reviewed and revised the themes until consensus was achieved. In the sixth and final phase—writing up—the first author was responsible for drafting the initial manuscript, which team members then reviewed and provided comments/suggestions.

The research team includes an older adult community member with work experience in emergency preparedness in a public health setting, a private industry expert in advocating for older adults, as well as doctoral candidates (including the first author), and professors who conduct research related to older adults, spanning multiple disciplines including law, health science, communications, and business. The principal investigator of the broader study (T.O.) has a research program focused on disaster resilience, specifically among high-risk populations. The first author (S.O.) is an OT with five years of clinical experience working with older adults in an acute care setting. This interdisciplinary collaboration allowed for thorough reflexivity throughout data collection and analysis, achieved through memoing and team meetings while discussing findings.

## Findings

The occupational participation of community-dwelling older adults in Canada during the COVID-19 pandemic was explored. A total of *n* = 67 older adults participated in the initial interview (denoted as “A”), with *n* = 37 participants from the same sample also completing a follow-up interview (“B”). Most participants conducted interviews in English, lived in a household with others in Ontario and identified as female, heterosexual, white, and living without a disability (Table 2). Most participants were under the age of 79, with the majority in the 70–79 age bracket for both interviews.

**Table 2:** Demographic Characteristics of the Sample Were Identified via Open-Ended Questions (not Pre-Defined Categories) and Organized by Initial and Follow-up Interviews (Denoted A and B, Respectively)

	A	B
Sample size	67	37
Preferred language		
English	59	36
French	8	1
Age		
60–69	22	13
70–79	36	21
80–89	8	2
90+	1	1
Gender		
Female	47	26
Male	20	11
Other	0	0
Sexual orientation		
Heterosexual	62	36
2SLGBTQ2I+	3	1
No answer or not disclosed	2	0
Canadian province of residence		
Ontario	50	31
Alberta	6	4
British Columbia	3	1
Quebec	1	0
Nova Scotia	1	1
No answer or not disclosed	6	0
Household make-up		
Living with others	46	28
Living alone	18	9
No answer or not disclosed	3	0
Racial background		
White	57	33
Black	3	1
Asian	2	1
Brown	1	1
No answer or not disclosed	4	1
Functional limitation identified		
No	57	32
Yes	6	3
No answer or not disclosed	4	2

**Figure 2.**
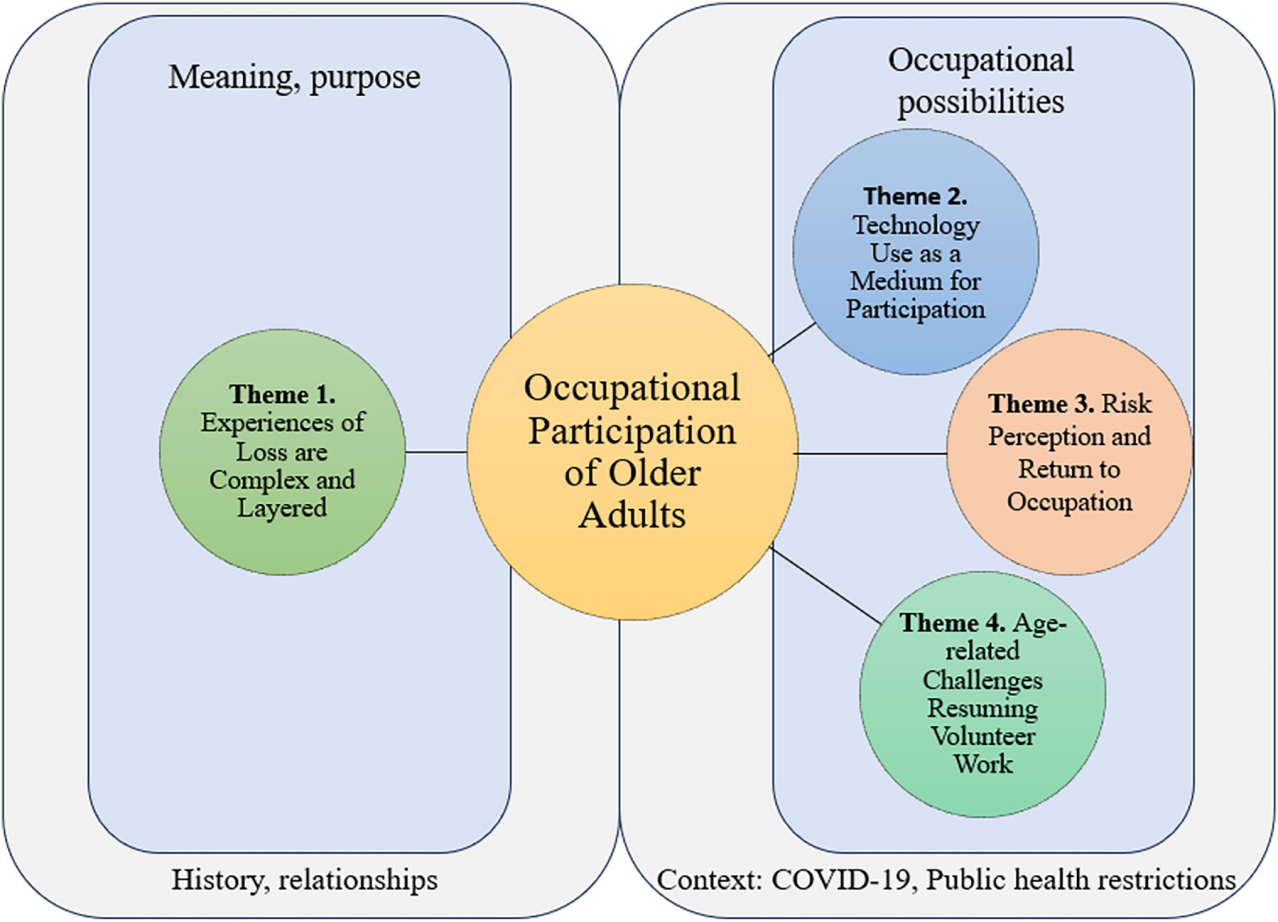
Thematic map using CanMOP (Egan & Restall, 2022) to contextualize the occupational participation of older adults and generated themes.

Using CanMOP to situate our understanding of occupational participation, we generated four themes with the two essential components of the model in mind (1—meaning and purpose and 2—occupational possibilities). We discuss the themes below and present corresponding participant quotations (denoted using a numerical pseudonym) from both data sets (A and B). The themes are displayed visually in a thematic map, contextualized using the CanMOP (Figure 2).

### Theme 1. Experiences of Loss are Complex and Layered for Older Adults

Many participants described leading vibrant, active lives prior to the pandemic. For example, through their experiences as volunteers in the community, balancing work with grandparent duties, participating in social clubs, engaging in physical activities, and participating in hobbies—from baking to attending the local theatre. As one participant described, the meaning associated with these activities was, “*Well, [they mean] everything, that's my life.” (P23 A)*. In the early days of the pandemic, restrictions abruptly ended many activities, altering daily routines and rituals. Participants described these changes as affecting their mood and motivation, namely they experienced more boredom and restlessness. In the following quotation, one participant highlights how the pandemic influenced her creativity.

*I have a grandson coming, I can’t get into a project for him. I’m excited for this event like you wouldn’t believe, but I have no - all my creativity has dried up. (P17 A)*

Not being able to do desired activities with others was articulated as a loss. Being deprived of relatedness in occupation was challenging for many participants who described joy and pleasure in the “doing” of occupations with others, and the comfort of shared experiences. For some, loss of relatedness in occupation impacted their ability to cope with evolving pandemic restrictions.

*That's the single biggest impact – that you’re not able to share any pleasures. You can certainly share misery over the phone, but there is nobody there to put an arm around you or to say ‘Come on over and we’ll have a coffee’ or ‘Let's go for a walk.’ The coping mechanisms that I have had have basically been taken away from me. (P37 A)*

The loss was also discussed in relation to aging and retirement, adding another layer of complexity to their experiences. Some participants highlighted how restrictions led to a sense of “lost” time, referencing the loss of years of their lives perceived as active and healthy.

*I'm in good health and everything, but how many years are we going to be able to travel and do lots of these things we want to do? And I feel kind of jipped a bit that I've lost a year and a half of travels. […] I really do feel like I've lost a year and a half out of the happy part of my life. (P42 A)*

Participants who identified themselves as “retired” from the workforce discussed how their perceptions of life during retirement had changed. During a time of life that can be marked by more free time and choice, participants highlighted how restrictions disrupted retirement ideals and led to a lack of spontaneity.

*Retirement has always been, for me, kind of a three-phase life experience, the first phase is go-go and second phase is slow-go and third phase is no-go. And with the pandemic, both my wife and I have experienced a slamming of the breaks where we were in the go-go phase and now it's been no-go. So, we kind of skipped over the slow-go completely. And that just is probably been the biggest impact. (P40 A)*

Collective experiences of loss appeared to stem from sudden changes to activities and routines that were disrupted by public health restrictions. Implications for mood, motivation, creativity, and relatedness, along with perceptions of “lost time” and disrupted retirement ideals. In our analysis, we situated these layered experiences of loss with the purpose and meaning participants ascribed to their occupations, which can be seen in Figure 2.

### Theme 2. Technology as a Medium for Occupational Participation

When in-person contact was restricted during the pandemic, many people turned to technology to access and participate in occupations. The use of technology was described for passive activities like watching television, videos, and streaming, and for active activities, like engaging in social groups, purchasing goods and services, and online learning opportunities. Related to accessibility, few participants described challenges with accessing the physical technology needed for digital participation, and this is likely a reflection of the sociodemographic characteristics of our sample. However, participants did highlight challenges they observed for the older adult demographic generally in gaining access to and having support to manage technology.

*…the other thing too is the cost of technology. I mean what I have to pay every month here just to keep up – because I do everything online now. I'm so happy about that and I have so much delivered to the door and I just think it's all a wonderful age for us as seniors but only if we're adept at using it all. And mostly, a lot of seniors […] they don't have access to that. I think there's a lack of empathy for seniors who aren’t up on [technology use]. (P60 B)*

For participants in this study, there was a range of experiences related to using technology as a medium for participation, from rejecting it to embracing it. The meaning and purpose of each unique activity seemed to dictate where participants fell on the continuum of technology use (rather than generalized to all activities). A few examples are provided below.

In this first example, we found that for certain individuals—such as musicians who perform in bands or sing in choirs—skill enhancement motivated their participation. As highlighted in the quotation below, technology was not seen as a satisfactory substitute for in-person engagement for these participants.

*A big thing for me is I am a musician of sorts and we have been unable to get together to work up any new programs or do recordings […] it's impossible to do it without fair expense online because of the delays involved in the transmission […] and you don't notice it when you're talking with someone, but if you try and play music together at a distance it doesn't work. There's no synchronization. So that's probably one of the biggest downers for me. (P52 A)*

Others identified social connection as their primary motivation for participation through technology. These participants often referred to technology use as “better than nothing” but not adequately supporting their need for social connection. “*It's really hard to not see people's expressions, to actually talk to them when you know you're seeing someone on [a virtual platform]. I mean, it's better than nothing, but […] it's just frustrating” (P45 A).*

Participants, like those quoted above (P52 A and P45 A), who were dissatisfied with participation via technology often expressed a desire to resume in-person activities as soon as possible. Their remarks hinted at technology use as transient and insufficient for sustaining their long-term needs for those activities.

*I would like to get back to the regular routine of meeting up with everybody on a regular basis, being able to go and play Mahjong, because it was mostly a social thing. I think there are people who do something like play, and it's partly social, but there are people who are passionate about playing the game. And we're not; it's more of just a social thing. (P13 A)*

While the previous examples center on occupation, individual factors also shaped participants’ attitudes toward technology. Some described their readiness to adopt technology in terms of openness or motivation to explore new ways of “doing.” Those who embraced technology explained how it created opportunities for participation that would have been impossible otherwise in the pandemic context. The two quotations below contrast different experiences of the spectrum of adopting technology use for participation.

*My [piano] teacher started to teach after two or three months [into the pandemic], […] and I just never really embraced learning online. Now I'm ready to, but I just can't seem to get my motivation mustered up (P44 A)*

*If not for [virtual platform], I don't know what I would have done. I've taken courses. I’ve done [participation in the social group], […] all my bridge is online. […] It's not real, but it's better than the alternative. (P45 B)*

All participants in this sample had experience with technology use, whether through active or passive participation. Their stance on embracing or rejecting technology as a medium for participation varied. Some linked their attitude toward technology to specific activities, while others related it to personal factors, like openness, willingness, or motivation to embrace and learn how to participate digitally. The implications for less “tech savvy” older adults was a concern as it could lead to lack of digital participation.

In Figure 2, we highlight this theme within occupational possibilities, noting that for some participants technology created new possibilities for participation while for others this was not the case. However, we also acknowledge that whether technology use was viewed as an acceptable alternative was strongly tied to the meaning and purpose participants ascribed to their occupations, for example, as meeting the need for relatedness or building competence with regards to a particular skill.

### Theme 3. Risk Perception Influences Return to Occupation

With the easing of public health restrictions, individuals gained greater flexibility in selecting the what, when, where, and with whom of occupation. While many expressed a desire to “return to normal” and resume their pre-pandemic routines, we discovered a complexity of factors influencing their perception of the risks associated with a return to participation. These factors, which ranged from micro to macro levels, shaped their views on the safety of returning to activities.

Risk factors at the micro level were described in terms of personal comfort level, subjective health status, and knowledge related to COVID-19. Many participants described making their assessment of risk based on their own perceptions and also in relation to people within their households and those they may come into contact with. In the community, many expressed greater comfort engaging with people and in organizations that shared similar rules or behavioural norms, “*Like going to the gym, if people are following the rules and the gym is following the rules, then I feel safe.” (P6 A)*

*I am 75, nearly 76 years old, and I do understand the statistics, and in my time of life I consider the risk to me versus the risk associated with doing the things that I want to do for the next few years of my life, is one I'm willing to take. (P41 B)*

*You have to be deliberate and cautious and think about [what] the risk tolerance of my friends will be. For instance, some have compromised immune systems or other life circumstances that might affect their risk tolerance. (P3 A)*.

Media messaging around older adults also shaped participants’ risk perceptions and decision-making around return to occupations. Specifically, emphasis on the vulnerability of older adults during the pandemic was highlighted and shaped behaviour. One participant indicated, “*I'm reminded constantly that I'm very vulnerable [through media news stories], and that […] I'm in the vulnerable population, and if I get it, I'll probably die. So that certainly has an impact on my behaviour” (P49 A).*

Other participants remarked how emphasis on the vulnerability of older adults played a role in conversations with family and friends, and the opinions they received from these important people in their lives. Some participants highlighted concerns about the potential impact on their autonomy around decision-making and return to occupation. For example, opinions from important others could influence the perceived accessibility of occupations regardless of the public health restrictions in place.

*The other thing that I want to stress, even though we’re older and it's COVID, I still safely want to maintain our independence as much as possible. I don’t want to rely on people if I don’t need to and at the beginning of this, I think we were all kind of scrambling and people were not even letting us go [grocery shopping], never mind if we wanted to or not. (P60 A)*

It was noted by many participants that news stories often intended to be compassionate sometimes failed to fully consider how restrictions impacted older adults, leading to ageism. For example, one participant pointed out that the adverse effects of public health restrictions on older adults were not receiving sufficient attention, “*I think there is a lot of ageism [in the media], I mean well-meaning maybe, but still, I don’t think it is good for older people to be so stuck in their homes.” (P4 A)*

Navigating risk perception was intricate, as evidenced by insights from participants in this sample. They emphasized a multitude of factors shaping their decision-making around return to occupations put on hold during the pandemic. Notably, ageism and the portrayal of older adults as vulnerable in media discourse also played a role. In Figure 2, this theme was placed within occupational possibilities to highlight how risk perception could shape occupational participation.

### Theme 4. Age-Related Challenges for Older Adults Resuming Volunteer Work

Many participants in our sample described participating in volunteer work in their communities prior to the pandemic. These activities abruptly stopped when initial pandemic-related restrictions were imposed. As restrictions gradually eased and organizations began to reopen, some participants navigated temporary policies based on age which restricted their return to volunteer work. The quotations below illustrate instances where participants experienced age-related challenges in attempting to resume in-person volunteer.

*Another volunteer job I did before [the pandemic], was both my husband and I, we volunteered at [a community centre] […] Of course, that all stopped, and when they started taking back volunteers, they wouldn’t take anyone over the age of 65, so we couldn’t go back. (P39 A)*

*There were times when [my volunteer work] stopped last year. They didn't allow anyone over 70 to come and volunteer so that probably stopped for, I would say maybe three or four months, and then I think they found that they needed their volunteers back. So that's when we got to do it. (P45 B)*

These temporary policies were described as well-intentioned—protecting older adults from illness—but contributed to experiences of loss, which were highlighted in theme 1, “*[Volunteering] made me feel so good. I miss contributing to things. I miss accomplishing things” (P42 A)*. Some participants shared how they adapted to continue contributing to their communities. For instance, in the quotation below, a participant explains how they shifted to volunteering in a different capacity. However, this adaptation led to reduced in-person contact, which did not necessarily offer the same level of satisfaction.

*I didn’t really mind that that was the rule [that people aged 65 and over couldn’t go back to volunteer in-person]. I could understand it, and now they allow us to deliver because we’re driving around in a car and just putting the boxes in front of the person's house or apartment building and waiting for them to come and get it. […] I used to say ‘I don’t want to just drive around, I want to really be with people’ so this is a different experience, you don’t really get to talk to people, you just wave at them. I think I prefer one-on-one contact when I’m with people. (P39 A)*

Volunteer work was a prominent example of a meaningful occupation impacted by pandemic restrictions. However, returning to this occupation was challenging for some participants due to age-related barriers. In Figure 2, we situated this theme within occupational possibilities to highlight challenges in the accessibility of this occupation. While alternative options were available in some instances, they didn’t always provide the same level of satisfaction, particularly for people whose purpose and meaning associated with participation was strongly tied to social connection.

## Discussion

In this study, we aimed to explore and understand the occupational participation of community-dwelling older adults during the COVID-19 pandemic. Our findings indicate older adults had complex and layered experiences of loss during the pandemic due to abrupt changes to daily routines. All participants had some exposure to technology as a medium for participation but there was a spectrum of experiences, from embracing to rejecting it. Upon the easing of public health restrictions, a multitude of factors were indicated in participants’ assessment of risk and return to occupations. Some participants who identified as volunteers encountered temporary age-related challenges in resuming this work.

Experiences of loss and occupational disruption during the pandemic are well documented. In this study, we described changes in mood, motivation, and creativity that coincided with loss. Similar Canadian studies on community-dwelling older adults conducted by Rotenberg et al. (2021) and Vesnaver et al. (2023) described similar changes that accompanied experiences of loss due to pandemic-related disruptions. However, these experiences are not unique to Canadian older adults, as researchers reported similar findings in countries like The Netherlands and Sweden, for example (Fristedt et al., 2022; Verhage et al., 2021). Unique to older adults were experiences of loss in relation to age. Participants in this study indicated perceptions of “lost time” or years of life where they felt they were in good health and able to participate in occupations they wanted to do, along with disrupted retirement ideals and a lack of spontaneity. Rotenberg et al. (2021) highlight how older adults felt “more aware” of their age during the pandemic and this was linked to the framing of the older adult demographic as high-risk during this time. Disrupted retirement ideals and spontaneity have been associated with changes to social and leisure activities during the pandemic (Koskinen & Leinonen, 2022; Sweeney & Zorotovich, 2021). This is concerning because these activities are important for fostering health and well-being as people age (United Nations [UN], 2020).

Loss of relatedness or social connection through occupational participation also contributed to feelings of loss for participants in this study. Participation through technology use, for example, was not always satisfactory for people who sought social connection. Similarly, older adults who described adapted volunteer work that was limited to options with minimal social contact did not always find this satisfactory if the purpose was to connect with others. Carlsson et al. (2022) highlight similar findings of older adults in Sweden who experienced a disrupted sense of belonging and loss of social connectivity. Rotenberg et al. (2021) also highlight how the quality of connection through technology was limited but still met some social needs of older adults. Many people turned to digital technology during the pandemic to meet needs like social connection. For older adults, social connectivity fostered through engagement in activities has been linked to maintaining cognitive function, supporting emotional well-being, and overall quality of life (Kelly et al., 2017; UN, 2020).

When it came to occupational participation through technology use, participants highlighted qualities beyond social connection which influenced whether they viewed it as an adequate alternative to in-person engagement. For example, for people focused on skill-building or developing competence related to music—like playing an instrument or singing with a group—they highlighted limitations with respect to technology use. These findings speak to reasons why older adults may be reluctant to adopt or sustain technology use for participation beyond well-documented challenges like technological accessibility and proficiency. Digital disengagement has been cited as concern for older adults, where they use but later abandon technology use for a variety of reasons (Olphert & Damodaran, 2013). With continued advancements and widespread use of technology, research indicates that older adults in particular may benefit from technology to optimize independence, social participation, and accessibility as they age (Fang et al., 2018; Gellis et al., 2012).

When pandemic restrictions were lifted or reduced, people were faced with decision-making about their risk perceptions and resuming previous in-person occupations. A unique consideration for older adults was the framing of risk and vulnerability which permeated media messages—and conversations with important others, like family and friends—and then influenced their behaviour. Emphasis on vulnerability was also apparent with temporary age-related restrictions limiting accessibility of volunteer work for older adults. Narratives of “vulnerability” within public COVID-19 discourse have been described as having detrimental effects by reinforcing negative stereotypes of older adults and fueling intergenerational friction based on perceived acceptable age-based behaviour (Ayalon et al., 2021; Fraser et al., 2020; Swift & Chasteen, 2021). Policies emphasizing the protection older adults have been described a paradox, for example, measures aimed at protecting older adults from COVID-19 illness and mortality have led to increased isolation and a decline in health (Lagacé et al., 2024; Rotenberg et al., 2021). This led to increased concerns about the autonomy of older adults within the pandemic context (Fuente et al., 2023; Rotenberg et al., 2021; Verhage et al., 2021). Viewing the COVID-19 pandemic through a disaster lens, it is essential to consider the ways in which social and colonial determinants of health intersect—and give rise to risk and “vulnerability”—rather than considering only one factor, like age (O'Sullivan & Phillips, 2019).

The findings from this study move beyond simply indicating that occupational participation was affected for older adults during the COVID-19 pandemic and delve deeper into *how* participation was influenced—for example, through technology use and risk perception. This nuanced exploration, facilitated by CanMOP (Egan & Restall, 2022), was a strength of this study. By understanding how the occupational participation of older adults was shaped by the pandemic, we can better inform and expand the role of OTs when similar events occur in the future. Shedding light on the purpose and meaning associated with occupational participation is essential to illuminate why technology, for example, may or may not be viewed as a satisfactory alternative to in-person engagement. While societal influences, like the framing of older adults in the media or organizational policies can shape occupational possibilities. Abrupt disruptions to daily routines can lead to experiences of loss. Knowing this, OTs can use their skillsets to foster participation and expand occupational possibilities for older adults in this context.

### Future Directions

Future research should explore how the occupational participation of older adults could be enhanced when future pandemics occur and subsequent public health measures are put in place. Involving older adults as stakeholders in this process may help to ensure a balanced perspective of what they identify as their needs and what assets they have to support their own resilience in this context. Further, future research should also consider the experiences of OTs who collaborated with older adults during the COVID-19 pandemic to better understand facilitators and barriers to effectively supporting this population. This will be crucial given the escalating frequency and severity of disasters like influenza pandemics worldwide, and the continued rise of population aging.

### Limitations

Several limitations are important to note for this study and how the findings are interpreted. The interview data across the two time-points were not explored using a longitudinal approach as the experiences captured did not reveal substantial differences in occupational participation, and this is likely due to the prolonged duration of the pandemic, surpassing initial expectations. The study sample had a limited representation of individuals over the age of 79 and lacked diversity in terms of race and gender. Further, related to technology use, participants in this study did not highlight challenges with accessibility—despite pointing to challenges for older adults generally—and this is likely indicative of the sociodemographic characteristics of the participants. A greater understanding of people who experienced compounding social vulnerabilities, such as gendered impacts and lower socioeconomic status, may provide further insights into the influence of pandemic-related restrictions, ageism, and other forms of discrimination on occupational participation.

## Conclusion

Occupational participation is essential for the health and well-being of people. In old age, participation in meaningful occupations has been linked to optimizing multi-dimensional aspects of functioning and quality of life. The COVID-19 pandemic led to occupational disruption, and older adults indicated unique experiences and a sense of loss linked to age. Broad social factors like the framing of older adults in the media and age-related restrictions challenged older adults’ participation during the pandemic. While technology use created occupational possibilities for some, it was not always adopted as a satisfactory alternative. The findings from this study underscore the importance of illuminating the meaning and purpose associated with participation in unique activities. The CanMOP was a helpful tool to understand the nuances underlying the participation of older adults in the pandemic context. OTs who work with older adults may benefit from these findings given the ongoing concerns about the frequency of future influenza pandemics, and other disasters.

## Key Messages

- The occupational participation of community-dwelling older adults was disrupted during the COVID-19 pandemic and participants described unique experiences linked to age.
- The CanMOP was used as a tool to explore occupational participation of older adults and lead to a nuanced understanding of how meaning and purpose is linked to participation, particularly technology use.
- Social influences, like the framing of old adults as vulnerable through the media and age-related restrictions challenged occupational participation during the pandemic.
